# Boys with conduct problems and callous-unemotional traits: Neural response to reward and punishment and associations with treatment response

**DOI:** 10.1016/j.dcn.2017.12.004

**Published:** 2017-12-15

**Authors:** Amy L. Byrd, Samuel W. Hawes, Jeffrey D. Burke, Rolf Loeber, Dustin A. Pardini

**Affiliations:** a University of Pittsburgh School of Medicine, Department of Psychiatry, United States; b Florida International University, Department of Psychology, United States; c University of Connecticut, Department of Psychological Sciences, United States; d Arizona State University, Department of Criminology, United States

**Keywords:** CP, conduct problems, CU, callous-unemotional, HC, traits healthy controls, SNAP, Stop-Now-And-Plan, Conduct problems, Callous-unemotional (CU) traits, Reward, Punishment, fMRI

## Abstract

Abnormalities in reward and punishment processing are implicated in the development of conduct problems (CP), particularly among youth with callous-unemotional (CU) traits. However, no studies have examined whether CP children with high versus low CU traits exhibit differences in the neural response to reward and punishment. A clinic-referred sample of CP boys with high versus low CU traits (ages 8–11; n = 37) and healthy controls (HC; n = 27) completed a fMRI task assessing reward and punishment processing. CP boys also completed a randomized control trial examining the effectiveness of an empirically-supported intervention (i.e., Stop-Now-And-Plan; SNAP). Primary analyses examined pre-treatment differences in neural activation to reward and punishment, and exploratory analyses assessed whether these differences predicted treatment outcome. Results demonstrated associations between CP and reduced amygdala activation to punishment independent of age, race, IQ and co-occurring ADHD and internalizing symptoms. CU traits were not associated with reward or punishment processing after accounting for covariates and no differences were found *between* CP boys with high versus low CU traits. While boys assigned to SNAP showed a greater reduction in CP, differences in neural activation were not associated with treatment response. Findings suggest that reduced sensitivity to punishment is associated with early-onset CP in boys regardless of the level of CU traits.

## 1 Introduction

Although childhood-onset conduct problems (CP) have been consistently associated with the development of severe and chronic antisocial behavior, many children who exhibit severe CP do not engage in severe delinquency during adolescence or adulthood (Moffitt, 1993; Byrd et al., 2012). A growing number of studies have found that callous-unemotional (CU) traits (e.g., lack of empathy and guilt) may help further delineate a subgroup of children with CP at heightened risk for exhibiting severe and persistent delinquency (Frick et al., 2013). As a result, CU traits have recently been added as a specifier for conduct disorder in The Diagnostic and Statistical Manual of Mental Disorders, Fifth Edition (DSM-5; American Psychiatric Association, 2013) and there is increasing interest in identifying unique etiological factors may underlie the development of CP among youth with CU traits.

Abnormalities in reward and punishment processing have long been implicated in the development of CP, particularly among youth with CU traits (Byrd et al., 2013; Blair et al., 2016; Hyde et al., 2013). Theoretically, researchers have suggested that a heightened sensitivity to reward and reduced sensitivity to punishment (i.e., loss of a desired stimulus or presentation of an unpleasant stimulus) increase risk for the development and persistence of CP. Behavioral studies have found that CP youth exhibit a greater affinity for large, immediate rewards using risk taking paradigms (Fairchild et al., 2009; Syngelaki et al., 2009; Schutter et al., 2011), and difficulty inhibiting a previously rewarded response in the face of increasing punishment during passive avoidance (Hartung et al., 2002) and response reversal paradigms (O'Brien et al., 1994; O'Brien and Frick, 1996; Matthys et al., 1998). Moreover, there is some evidence to suggest that these deficits are most pronounced among CP youth with high CU traits (Budhani and Blair, 2005; Frick et al., 2013, Frick et al., 2003; Byrd et al., 2013). However, these studies assess ‘overall performance’ using behavioral tasks that include aspects of both reward *and* punishment processing, limiting our ability to disentangle whether the observed performance differences are due to abnormalities in processing reward, punishment, or both. Additionally, it is unclear whether CP youth exhibit deficits in processing reward and punishment at a particular stage of learning (e.g., initial encoding/receipt, acquisition, extinction) or across multiple stages (Balsam et al., 2010).

Over the last several years, neuroimaging studies have attempted to address these limitations by disaggregating the neural response to reward and punishment across various stages of learning. Some evidence indicates that, relative to healthy controls (HC), youth with CP exhibit functional abnormalities in regions associated with reward processing (i.e., ventral and dorsal striatum), punishment processing (i.e., amygdala), and higher-order regulatory function (i.e., medial prefrontal cortex, mPFC; anterior cingulate cortex, ACC) (for reviews see Byrd et al., 2013; Hyde et al., 2013; Blair et al., 2016; Alegria et al., 2016). This altered functional activation has been documented using tasks involving either reward or punishment anticipation and/or receipt (Bjork et al., 2010; Cohn et al., 2015b, Cohn et al., 2013) as well as tasks incorporating aspects of both reward and punishment during acquisition and/or extinction (Finger et al., 2008, Finger et al., 2011; White et al., 2013, White et al., 2016; Crowley et al., 2010; Cohn et al., 2015a). While prominent theory posits that a hypersensitivity to reward and a hyposensitivity to punishment underlies the development of CP and CU traits (Newman and Wallace, 1993; Frick et al., 2014), the neuroimaging literature is not entirely consistent, with noted discrepancies in directionality of results (i.e., hyper- versus hypo-activation) (Byrd et al., 2013; Blair et al., 2016; Hyde et al., 2013). Though inconsistencies may be associated with task-specific differences or an inability to completely disambiguate responsivity to reward and punishment processing at individual stages of learning (Richards et al., 2013), additional limitations regarding sample heterogeneity may also obscure findings.

Many studies in this area have focused on functional differences in reward/punishment processing between heterogeneous groups of CP youth and healthy controls (e.g., Rubia et al., 2008; Bjork et al., 2010; Crowley et al., 2010), potentially obfuscating important etiological differences. Those studies that have assessed CU traits report mixed findings and this may be attributable to an extreme group approach (i.e., CP youth with high CU traits versus healthy controls; Finger et al., 2008, Finger et al., 2011) or suppressor effects arising from a failure to account for unique associations between CP versus CU traits and variation in neural response to reward/punishment (see Cohn et al., 2015a, Cohn et al., 2013). Additionally, these studies have focused almost exclusively on adolescence, a developmental period characterized by substantial changes in the neural circuitry underlying reward and punishment processing (Steinberg and Morris, 2001). Thus, the current study sought expand on previous research by focusing on potential differences in reward/punishment processing *between* subgroups of pre-adolescent youth with CP and high versus low CU traits.

### 1.1 Implications for intervention

The examination of reward and punishment processing among subgroups of youth with CP is particularly important from an intervention perspective. Although multimodal interventions that include child-focused cognitive-behavioral therapy (CBT) and parent management training (PMT) are generally effective at reducing CP among children (Webster-Stratton et al., 2004), it is well-documented that these interventions are not equally effective for all youth (Hawes et al., 2014; Matthys et al., 2012). Some have suggested that CP youth with CU traits may be more responsive to reward-based intervention and more resistant to punishment focused strategies (Hawes et al., 2014). However, we are aware of no existing studies that have examined whether functional abnormalities in reward and/or punishment processing are associated with treatment response among CP youth with high versus low CU traits.

### 1.2 Current study

To address noted gaps in the literature, the current study used functional magnetic resonance imaging (fMRI) to assess neural responsivity to the receipt of reward and punishment among pre-adolescent boys with CP and varying levels of CU traits, and HC. To examine potential differences *between* CP boys with high versus low CU traits, group-based analyses were used. Additionally, CP and CU traits were examined dimensionally in continuous analyses. Consistent with theory and prior research, we hypothesized that CP would be associated with reduced sensitivity to punishment and greater sensitivity to reward as evidenced by decreased amygdala activation to punishment, increased striatal activation to reward and reduced activation in the mPFC and ACC to both reward and punishment. Moreover, we hypothesized that these neural abnormalities would be most pronounced in those boys with CP and high CU traits. Finally, in exploratory analyses, this study examined whether abnormalities in the neural correlates of reward and/or punishment processing predicted post-treatment levels of CP following random assignment to an empirically supported multi-modal intervention (i.e., Stop-Now-And-Plan; SNAP).

## 2 Methods

### 2.1 Participants

Participants were 64 boys 8- to 11-years-old (*M* = 10.68; *SD* = 1.18): 37 boys exhibiting CP and 27 matched HC. CP youth were recruited from a larger treatment study (Burke and Loeber, 2014) and deemed eligible if they presented with clinically significant behavior problems (i.e., externalizing composite T-score > 64; aggressive behavior, rule breaking, conduct problems subscale T-scores >70) according to the Child-Behavior Checklist (CBC-L; Achenbach, 1991) For further details on inclusion and exclusion of CP youth, see Burke and Loeber (2015).

HC were recruited predominantly from local pediatricians’ offices in the community and matched as a group to CP youth on age and race. Inclusion criteria necessitated problems below the at-risk threshold on all externalizing and internalizing scales of the CBCL (T-score < 60). All procedures were reviewed and approved by the Institution Review Board. Written informed consent was obtained from parents/guardians and youth provided assent prior to each assessment.

### 2.2 Procedure

All CP youth and HC controls completed a baseline assessment, which included measures of CP, CU traits and covariates (e.g., demographics, IQ). Eligible CP and HC youth also completed an fMRI scan. Following the fMRI scan session, CP youth were randomly assigned to one of two treatment conditions: 1) a multimodal CBT/PMT intervention (i.e., SNAP; n = 21) or 2) standard services (SS; n = 16) in the community as a part of the larger treatment study (see Burke and Loeber, 2015). Finally, CP youth were reassessed 3-months later, after treatment was completed. Due to attrition, post-treatment data was only collected on 34 CP boys (19 assigned to SNAP; 15 assigned to SS). For review of the larger intervention, see Burke and Loeber (2015).

### 2.3 SNAP intervention

The SNAP program is an empirically supported, manualized intervention and takes a multimodal approach by focusing on two core components: 1) child CBT groups emphasizing self-control skills and problem-solving techniques; 2) parent PMT groups focused on behavioral strategies for consistent reward and punishment implementation. Groups use modeling, behavioral rehearsal/role plays and home practice exercises and are offered simultaneously for 90-min for 12 consecutive weeks. For further details on this intervention see Augimeri et al. (2007).

### 2.4 Standard services

Participants who were assigned to the SS condition received assistance from project staff in their efforts to engage in treatment services, with a focus on securing evaluations to determine eligibility for wraparound services available in the local community (i.e., ∼10 service hours per week). Despite the high level of behavioral problems shown by participants, clinical evaluations conducted by community providers did not always result in recommendations for wraparound services and, in some instances, recommendations were made for less intensive service options. Of those assigned to standard services, only 2 children (13.3%) were engaged in wraparound services and 5 children (33.3%) engaged in lower intensity mental health services by the 3-month follow-up assessment. This was comparable to percentage of youth engaged in the larger treatment sample (Burke and Loeber, 2015). For further details, see Burke and Loeber (2015).

### 2.5 Measures

#### 2.5.1 Child-Behavior checklist (CBCL)

The CBCL is a 113-item parent-report questionnaire that assesses emotional and behavioral problems in children (Achenbach, 1991). Scores on the DSM-oriented conduct problem subscale (17 items) were used in the current study. Internal consistency for the CP subscale at baseline and follow-up ranged from excellent to good (α = 0.93; α = 0.83).

#### 2.5.2 Antisocial process screening device (APSD)

The parent- and youth-reported APSD (Frick and Hare, 2001) contains 4-items (i.e., lack of remorse or guilt, lack of empathy, unconcerned about performance, and shallow or deficient affect) that formed the basis for the DSM-5 specifier for CD referred to as “with limited prosocial emotions”(American Psychiatric Association, 2013). Parent- and youth rated these 4-items on a 3-point Likert scale (0 = ‘not true’ to 2 = ‘very true’) and parent- and child-report was combined across the two informants by taking the higher of the two ratings for each item. Internal consistency for the CU subscale was acceptable (α = 0.70).

### 2.6 Group assignment

CP youth were divided into subgroups based on high versus low CU traits, as measured by the 4-items described above. Those items scored a 2 (‘very true’) by either parent- or youth-report were characterized as ‘present’. CP youth with the presence of at least two of the four items were classified as having high CU traits, consistent with DSM-5 criteria for the conduct disorder specifier (American Psychiatric Association, 2013). This resulted in 24 boys with CP and low CU (‘CP only’) and 13 boys with CP and high CU (‘CPCU’).

### 2.7 Covariates

Age, race, receipt of public assistance, and IQ as well as ADHD and internalizing symptoms were included as covariates considering research documenting consistent associations with CP (Loeber and Keenan, 1994; Waschbusch, 2002).

#### 2.7.1 Earlscourt family information form

Parents reported on basic demographic information, including age, race (dichotomized Caucasian = 0, African-American = 1), and receipt of public assistance (dichotomized no = 0; yes = 1).

#### 2.7.2 Kaufman brief intelligence test-2 (KBIT-2)

The KBIT-2 (Kaufman and Kaufman, 2004) is comprised of two subscales (i.e., verbal and non-verbal intelligence) that were combined to provide a composite score indicative of overall IQ.

#### 2.7.3 Child-Behavior checklist (Achenbach, 1991)

Parents reported on the ADHD subscale (8 items) and internalizing composite scale (32 items) at baseline. The internal consistency of these scales was good (α = 0.88, α = 0.83).

### 2.8 Card guessing task

**Fig. 1:**
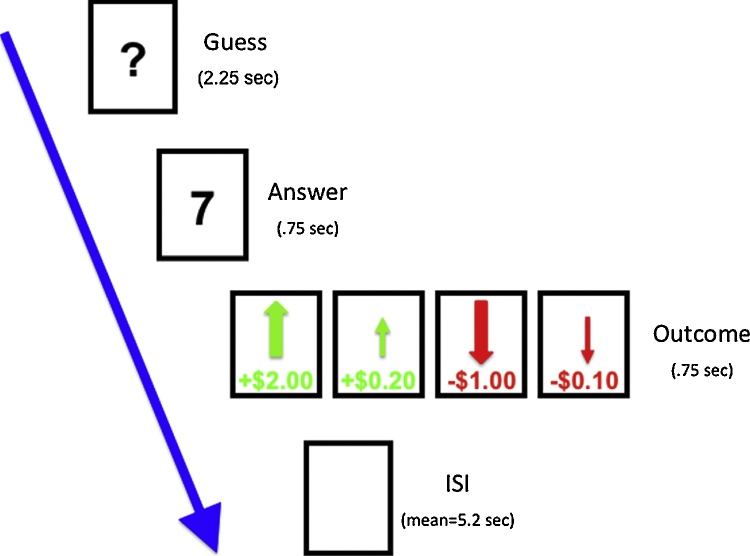
Schematic of events within each trial of the fMRI reward/punishment task.

Participants completed an adapted version of an event-related task designed to assess reward and punishment processing (Delgado et al., 2000). Boys were told they would be playing a card guessing game with the goal of winning as much money as possible. They were presented with a card that had an unknown value from 1 to 9, and were instructed to guess whether the value of the card was higher or lower than 5 by pressing one of two buttons (Fig. 1). The actual number was then presented, followed by a green arrow indicating whether they won a big ($2.00) or little ($0.20) monetary reward, or a red arrow indicating a big ($-1.00) or small ($-0.20) monetary loss (750 ms). The outcome of each trial was predetermined and presented in a fixed, pseudorandom order with a jittered inter-trial interval. A total of 10 trials per condition were presented within each 4-min and 10 s run and a total of 4 runs were presented. Boys practiced the task prior to entering the scanner and were paid $20 in “winnings” after completing the scan.

### 2.9 Neuroimaging procedures

Functional and structural images were collected using a Siemens 3T Magnetom TIM Trio. A high resolution anatomical image covering the entire brain was acquired using an axial 3D MPRAGE sequence, parallel to the AC-PC line (TE/TI/TR = 3.29 ms/900 ms/2200 ms, flip angle = 9°, 1 mm^3^ voxel, 192 axial slices, matrix size = 256 × 192). Functional images were acquired while participants completed a card guessing task (described below) using a gradient echo EPI sequence that covered 37 axial (AC/PC aligned) slices encompassing the cerebrum and most of the cerebellum (TR/TE = 2000/28 ms, FOV = 200 × 200, matrix = 64 × 64, flip angle = 90°; 3.1 mm^3^, 0 mm gap).

### 2.10 Image processing

All fMRI data was preprocessed using SPM5 (http://www.fil.ion.ucl.ac.uk/spm/software/spm5/). Functional images were first motion corrected using a two-pass procedure with sinc interpolation and these motion parameters were included as covariates of no interest in all analyses. After realignment, the structural MPRAGE image was coregistered to the corrected mean functional image and transformed into Montreal Neurologic Institute (MNI) stereotactic space. Warping parameters from this procedure were applied to the functional images to transform them into MNI space. Lastly, the functional images were spatially smoothed using a 6 mm full-width at half-maximum (FWHM) Gaussian kernel.

### 2.11 fMRI data analysis

For each subject, the blood oxygen level dependent (BOLD) response to the four conditions of interest (receipt of a big reward, little reward, big punishment, little punishment) was modeled separately for each run using a canonical hemodynamic response function. Covariates of no interest included the six motion parameters generated from the realignment pre-processing procedure. Separate intercept values were estimated for each functional run as an implicit baseline.^1^ Beta coefficients representing the average height of the BOLD response across the runs to each outcome of interest were used in subsequent group level analyses.

First, group differences in the BOLD response to the receipt of reward and punishment were examined using a 3 × 4 ANOVA, with group (HC, CP only, CPCU) entered as a between-subject factor and condition (big reward, little reward, big punishment, little punishment) entered as a within-subject factor. Second, regression analyses were conducted to examine associations between continuous CP and CU scores and BOLD response to reward and punishment. Bivariate associations between 1) CP and BOLD response to reward and punishment and 2) CU traits and BOLD response to reward and punishment were examined as well as multivariate associations (CP and CU traits entered simultaneously) with BOLD response to reward and punishment.. All associations were examined before and after statistically controlling for covariates.

All voxel-based analyses were initially tested within anatomically-defined regions of interest (ROIs), which included the amygdala, striatum, ACC and mPFC (i.e., BA10). ROI masks were defined bilaterally and generated using automated anatomical labeling (AAL) masks from the Wake Forest University (WFU) Pick-Atlas Tool (v3.0.3), with the exception of the striatum.^2^ To correct for multiple comparisons, a cluster-level significance threshold was delineated for each ROI using the Monte Carlo simulation program 3dClustim.^3^ Significant findings were further examined by calculating the average voxel-level BOLD value (i.e., betas) within an identified cluster for each individual and importing this data into SPSS.

### 2.12 Treatment outcome analysis

A final set of exploratory analyses were conducted to assess whether any of significant clusters identified in primary group analyses described above predicted CP severity at the 3-month follow-up assessment among boys assigned to the SNAP or SS treatment condition. This was done using a repeated-measures analysis of variance (ANOVA). Predictors in this model included one within-person factor (“time”) to model within-individual differences in CP from baseline (coded “0”) to the 3-month post-treatment assessment (coded “1”) and two between-person factors representing 1) treatment condition (SS = 0; SNAP = 1); 3) and 2) the BOLD response. A separate repeated-measures ANOVA was conducted for each of the significant clusters identified in the primary group (pre-treatment) analyses described above.

## 3 Results

### 3.1 Sample characteristics

The means and standard deviations for all study variables are presented in Table 1. All groups were equivalent regarding age, race and receipt of public assistance; however, groups differed slightly regarding IQ. HC had higher IQ scores than CP only youth, though there were no differences between CPCU youth and either of the other groups. As expected, CP only and CPCU youth had higher levels of CP, CU, ADHD and internalizing symptoms relative to HC. CP only and CPCU youth only differed on levels of CU traits.

**Table 1:** Means and standard deviations for all study variables by group.

	HC		CP only		CPCU	
	n = 27		n = 24		n = 13	
	M/%	SD	M/%	SD	M/%	SD
Age	10.46 ^a^	*1.24*	10.82 ^a^	*1.22*	10.83 ^a^	*0.89*
Race (African-American)	78% ^a^	–	88% ^a^	–	85% ^a^	–
Public Assistance (yes/no)	63% ^a^	–	54% ^a^	–	77% ^a^	–
IQ	100.63^a^	*15.52*	90.29^b^	*11.30*	96.23^a,b^	*10.69*
ADHD symptoms	50.56^a^	*1.31*	67.29^b^	*8.80*	68.15^b^	*7.57*
Internalizing symptoms	51.37^a^	*3.19*	63.96^b^	*6.97*	62.08^b^	*11.72*
Conduct Problems┼	51.00^a^	*2.04*	76.67^b^	*7.70*	77.00^b^	*8.62*
APSD CU Traits	3.96^a^	*1.58*	5.96^b^	*1.27*	9.38^c^	*1.33*
APSD Total Score	10.85^a^	*4.79*	21.33^b^	*4.38*	25.85^c^	*5.11*

*Note*. ADHD = attention deficit hyperactivity disorder; APSD = Antisocial Process Screening Device; CP = conduct problems; CU = callous-unemotional; HC = healthy controls; IQ = intelligent quotient. Means designated with different subscript letters are significantly different from each other (*p *<  .05) based on post-hoc independent sample *t*-tests.

┼ CP only and CPCU youth were also equivalent on aggressive behaviors subscale, rule breaking subscale and externalizing composite (*p *> .50) and both groups evidenced significantly greater scores than HC (*p < *.05).

### 3.2 Behavioral data analysis

Task performance differed slightly between groups. CPCU youth evidenced slower reaction times relative to CP only (*t*(35) = 3.32, *p <* .01) and HC (*t*(38) = −2.14, *p *< .05). Additionally, CPCU youth had more ‘non-responses’ relative to CP only (*t*(35) = −2.17, *p *< .05) and HC (*t*(38) = −2.84, *p *< .05). All groups responded to more than 85% of trials (n > 105 trials out of 120 trials), with each participant responding to at least 80% of trials in each condition (n > 24 trials out of 30 trials).

### 3.3 Task activation to reward and punishment

**Fig. 2:**
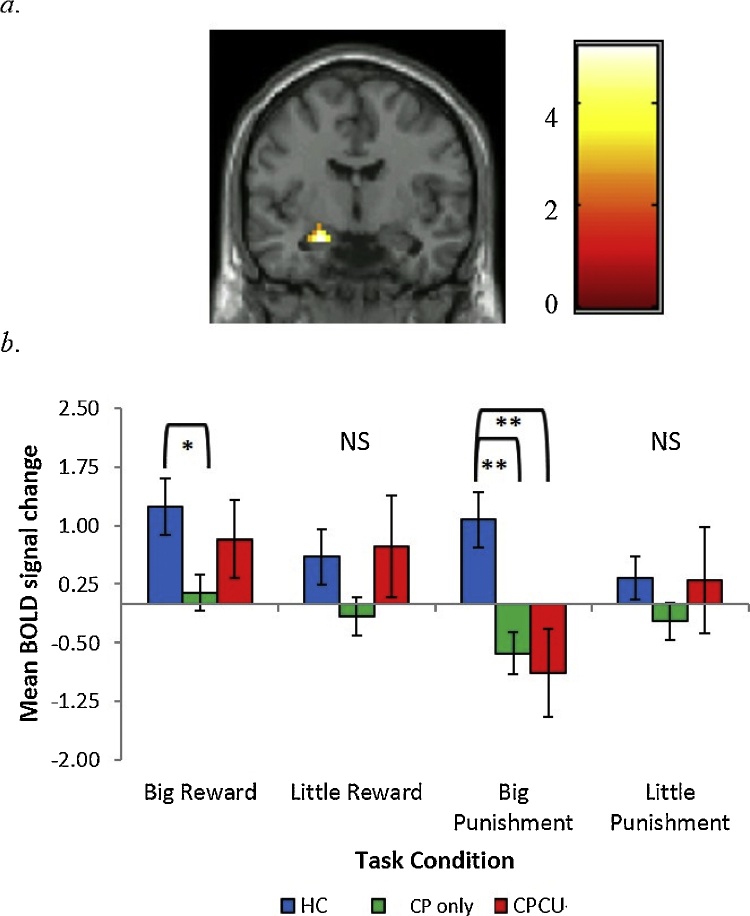
Interaction between group and task condition in the left amygdala. *a*. Region in the amygdala significant at *p < *.05, corrected using 3DclusterSim threshold for contiguous voxels (*F*(6,244) = 9.41, 22 voxels). Coronal slice shown at y = −6 (MNI peak voxel). Color bar reflects t-values; *b*. Bar graphs depict extracted mean BOLD signal change (%) across all voxels within the cluster along with standard errors. * = *p < *.05; ** = *p <* .01. *P*-values are based on Games-Howell pairwise comparisons for extracted mean BOLD response. *Note*. BOLD = blood oxygen level dependent; CP = conduct problems; CU = callous-unemotional traits; HC = healthy control; NS = non-significant.

Prior to examining potential group differences, preliminary analyses were conducted to examine task-specific, whole-brain activation to the receipt of reward and punishment (Supplemental Tables 1 and 2). The average BOLD response to reward and punishment across all participants indicated that the task produced robust activation throughout expected reward- and punishment-related circuitry (Supplemental Fig. 1, Fig. 2). Similar patterns of activation have been demonstrated in previous work using versions of this task and comparable tasks (e.g., MID) in healthy and clinical samples (e.g., Lutz and Widmer, 2014; May et al., 2004; Rubia et al., 2009).

### 3.4 Main effect of outcome

A main effect of outcome was observed in all ROIs (Table 2). All regions had greater activation during reward relative to punishment outcomes, and both were significantly greater than baseline.

**Table 2:** Significant suprathreshold clusters from 3 × 4 ANOVA

			Peak Coordinates (MNI)				
Brain region	R/L	Voxels	X	Y	Z	F	p-value
Main Effect of Outcome							
Amygdala	L	47	−16	0	−15	17.25	<0.001
Amygdala	R	38	19	0	−12	14.97	<0.001
Striatum	R/L	1000	16	9	−6	36.75	<0.001
ACC	R/L	503	−3	41	6	14.01	<0.001
mPFC	R/L	104	−3	53	−3	15.30	<0.001

Main Effect of Group							
mPFC	L	8	8	−6	50	7.63	0.001
Amygdala	L	14	−28	0	−22	5.64	0.004

Group X Condition Interaction							
Amygdala	L	22	−22	−6	−19	9.41	<0.001
Caudate	L	14	−12	3	15	4.37	<0.001

*Note*. R = right; L = left; ACC = anterior cingulate; mPFC = medial prefrontal cortex; OFC = orbital frontal cortex; BA = Brodmann’s area.

### 3.5 Main effect of group

A main effect of group was found in the left amygdala and mPFC (Table 2). Collapsed across conditions, CP only youth showed reduced activation in these regions relative to HC; however, these findings were reduced to trend level significance after controlling for covariates.

### 3.6 Group-by-condition interaction

A significant group-by-condition interaction was found for a cluster of voxels in the left amygdala (Table 2; Fig. 2). Further examination of this interaction indicated that both CP groups exhibited decreased amygdala activation following the receipt of punishment relative to HC. Group differences remained significant even after controlling for all covariates. Additionally, the CP only group exhibited lower activation to big reward in the left amygdala relative to HC. However, this was reduced to non-significance after accounting for covariates. There were no significant differences between CP groups in this region.

A significant group-by-condition interaction was also found for a cluster of voxels in the left caudate (Supplemental Fig. 3). Further probing of this interaction revealed that the CP only group exhibited a reduced activation to the receipt of a big reward relative to the CPCU and HC groups. Within this region, the CP only group also exhibited reduced activation to big punishment relative to HC. However, these group differences were reduced to non-significance after controlling for all study covariates.^4^

### 3.7 Continuous analyses

**Fig. 3:**
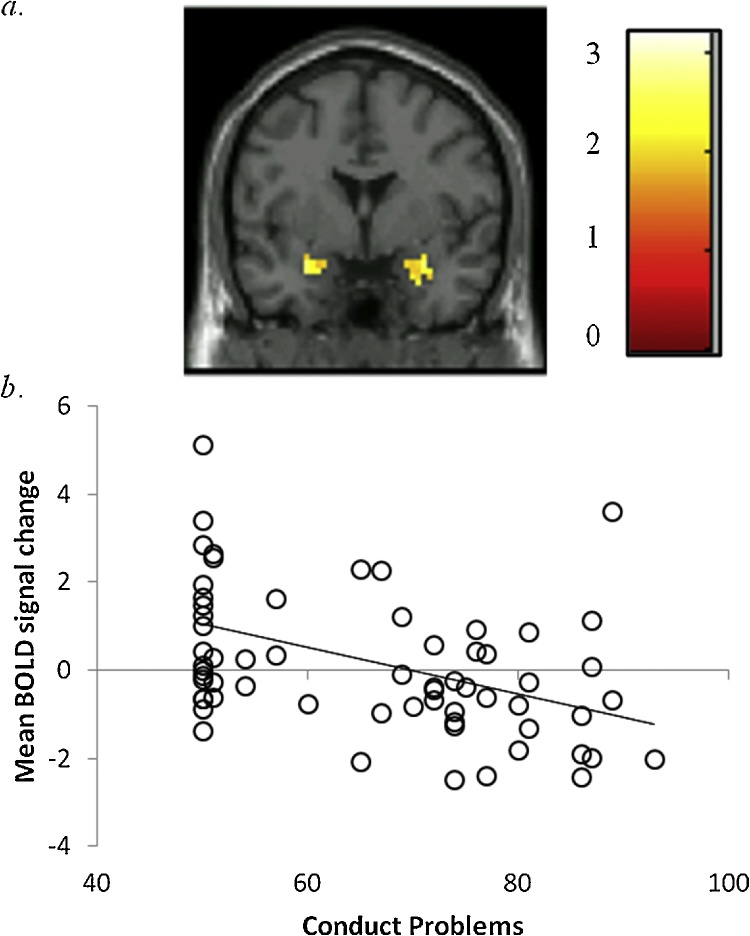
Activation in the bilateral amygdala is negatively associated with conduct problems, even after controlling for variance associated with callous-unemotional traits. *a*. Region in the bilateral amygdala significant at *p < *.05, corrected using 3DclusterSim threshold for contiguous voxels (Right amygdala: 33 voxels, t = 3.20; Left amygdala: 29 voxels, t = 2.87). Coronal slice shown at y = 0. Color bar reflects t-values; *b*. Scatter plot depicts association between baseline levels of conduct problems (x-axis) and mean BOLD signal change (%) in the amygdala (y-axis) after controlling for co-occurring callous-unemotional traits (*r* = −0.42). *Note*. BOLD = blood oxygen level dependent

Continuous associations with CP and CU traits were examined and findings mirrored group-based analyses (Table 3). Regarding punishment, CP was associated with reduced activation in the bilateral amygdala, even after accounting for CU and covariates (Fig. 3). Regarding reward, CP was uniquely associated with reduced activation in the left amygdala and CU was uniquely associated with increased activation in the right caudate (Supplemental Fig. 3); however, findings were reduced to non-significance after accounting for covariates.

**Table 3:** Suprathreshold clusters associated with CP and CU severity: Univariate and multivariate regressions.

				Peak Coordinates (MNI)				
	Brain Region	R/L	Voxels	X	Y	Z	z-score	p-value
Bivariate Associations								
Big Reward								
CP (- association)	Amygdala	L	20	−22	−3	−19	2.61	0.005
Big Punishment								
CP (- association)	Amygdala	R	46	34	3	−22	4.22	0.000
	Amygdala	L	40	−28	0	−22	3.88	0.000
	Striatum	R/L	279	3	−3	−12	3.99	0.000
	ACC	R/L	178	0	31	−6	3.73	0.000
	mPFC	L	23	−3	53	−6	3.28	0.001
CU (- association)	Amygdala	L	26	−28	0	−25	3.75	0.000
	Amygdala	R	25	34	3	−22	3.00	0.001
	Caudate/Putamen	L	16	−19	19	9	2.99	0.001
Unique Associations								
Big Reward								
CP controlling for CU (- association)	Amygdala	L	13	−19	−3	19	2.77	0.004
CU controlling for CP (+ association)	Caudate	R	13	19	−12	25	3.45	0.000
Big Punishment								
CP controlling for CU (- association)	Amygdala	R	33	34	3	−22	3.06	0.001
	Amygdala	L	29	−22	−6	−15	2.77	0.003

*Note*. R = right; L = left; CP = conduct problems; CU = callous-unemotional traits; + = positive; − = negative; mPFC = medial prefrontal cortex.

### 3.8 Treatment effects

**Fig. 4:**
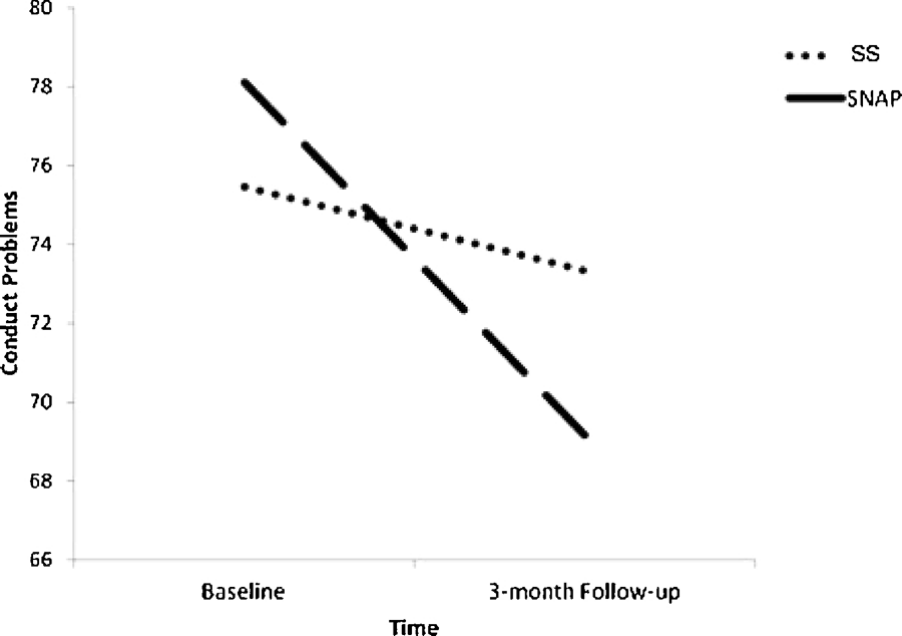
Significant reduction in conduct problems for youth participating in the SNAP intervention. Post-hoc paired sample *t*-tests revealed a significant reduction in conduct problems between baseline levels and 3-month follow-up for those youth participating in SNAP (*t*(33) = 5.14; *p *< .001). No differences were seen between conduct problems at baseline and 3-month follow-up for those youth in Standard Services (*t*(33) = 1.13; *p *> .25). *Note*. SS = Standard Services; SNAP = Stop-Now-and-Plan Intervention.

Three separate repeated measures ANOVA were conducted for each of the clusters that differentiated groups in the analyses described above (i.e., 1) left mPFC; 2) left amygdala; 3) left caudate; see Supplemental Table 4). There was no effect of BOLD response on treatment outcome, nor was there a significant interaction between BOLD response and intervention.^5^ However, there was a significant effect of intervention, as indicated by a significant time X intervention interaction (Fig. 4). Post-hoc paired sample *t*-tests revealed a reduction in CP from baseline to 3-month follow-up for youth participating in SNAP, whereas youth in the SS group did not experience change in CP across this same period.

## 4 Conclusions

This study sought to expand on previous research by examining neural response to the receipt of reward and punishment among CP boys with high versus low CU traits. Consistent with hypotheses, results provided evidence of reduced amygdala activation to punishment among boys exhibiting early-onset CP. Importantly, findings were consistent across distinct types of analyses (i.e., group-based versus continuous), and after accounting for confounds (e.g., race, IQ, ADHD). Results were notably less robust regarding reward sensitivity and group differences within the caudate and mPFC were reduced to non-significance after controlling for covariates. Noteworthy, this study failed to support the hypothesis that reward and/or punishment sensitivity is uniquely characteristic of CP youth with high CU traits. Moreover, exploratory analyses found no association between neural response to reward and/or punishment and treatment outcome, though there were greater reductions in CP among boys assigned to SNAP.

Regarding punishment sensitivity, findings from the current study are in line with prior neuroimaging research demonstrating abnormalities in amygdala structure and function among individuals exhibiting early-onset CP (Hyde et al., 2013, Hyde et al., 2016; Pardini et al., 2014). This reduced sensitivity to punishment has been well-documented among CP youth across childhood and adolescence (see Byrd et al., 2013; Alegria et al., 2016) and may hinder the development of conditioned associations between punishment and distress, ultimately increasing the likelihood of the development and persistence of CP (Kochanska, 1994). Indeed, recent work suggests these abnormalities are present as early as 3 years of age and serve to predict criminal offending in adulthood (Gao et al., 2010). Moreover, other research within this population notes abnormalities in amygdala reactivity during emotion processing (see Blair et al., 2016; Hyde et al., 2013), suggesting amygdala dysfunction likely contributes to a variety of behavioral deficits that may underlie the development and maintenance of CP.

The current study failed to support theory and behavioral research suggesting these deficits are specific to or most pronounced among CP youth with high CU traits. It should be noted that this is consistent with some previous work in this area, which has failed to find significant associations with psychopathic traits (broadly defined) and abnormalities in reward/punishment processing (e.g., White et al., 2013, White et al., 2016). This is not to suggest that these deficits are not characteristic of youth with CP and CU traits; instead, results highlight that these abnormalities may be better understood as a general dysfunction among youth with severe, early-onset CP. Additionally, it is important to consider that abnormalities in punishment (or reward) processing may be more pronounced during various stages of learning and that these deficits may be most evident during critical developmental periods. As such, continued work in this area is needed.

Results for reward processing were also inconsistent with hypotheses. Findings indicated that CP only youth exhibited reduced activation within the caudate, while CP youth with high CU demonstrated normative or slightly heightened activation within this region. Previous research has detailed notable inconsistencies regarding hyper- versus hypo-activation within reward related circuitry (Rubia et al., 2009; Bjork et al., 2010) and this may be related many factors, including variation in task design. As alluded to above, recent studies examining reward processing among youth with psychopathic features and/or CU traits have also produced mixed findings (see Blair et al., 2016), further underscoring the need for continued research in this area. Accounting for co-occurring internalizing problems may be particularly important when assessing reward processing, given evidence of reduced reactivity to reward among youth suffering from depression (Forbes et al., 2007). In this regard, it is noteworthy that group differences in reward sensitivity were reduced to non-significance after accounting for confounds (e.g., internalizing problems). Finally, it is also possible that reward processing deficits are most evident in the presence of competing reward and punishment stimuli (Newman and Lorenz, 2002).

Both CP groups evidenced lower activation across all reward and punishment conditions in the mPFC, a region responsible for top-down regulatory function over subcortical regions linked to reward and punishment processing (Cardinal et al., 2002). Although this is consistent with previous work in this area (see Byrd et al., 2013; Blair et al., 2016), it is noteworthy that decreased activation was only significant for the CP only group and findings were reduced to trend level significance after accounting for covariates. It is possible that our null findings may be related to the developmental timing of the current study (i.e., pre-adolescence), when differences in regulatory function may be less pronounced (Bjork and Pardini, 2015). Additionally, functional regulatory deficits may be most pronounced at later stages of learning (i.e., acquisition, extinction) when greater cognitive control is needed.

Importantly, neural abnormalities were unrelated to treatment outcome and failed to moderate the effectiveness of an empirically-supported multi-modal intervention. While these analyses were notably exploratory in nature given the small sample, results emphasize the utility of this intervention in youth with early-onset CP and mirror findings from the larger treatment study (Burke and Loeber, 2014). At the same time, intervention efforts typically boast effect sizes that are in the small to moderate range (Matthys et al., 2012). This may be related to the ‘one size fits all’ approach and a failure to assess and treat child-specific deficits at an individual level. Efforts to tailor interventions to meet child-specific needs may increase the effectiveness of social learning-based interventions (Dadds and Salmon, 2003; Hawes et al., 2014). Moreover, an examination of how treatment induced changes in child and/or parenting behaviors serve to interact with identified deficits in reward and/or punishment processing may help to further elucidate more complex moderation mechanisms.

### 4.1 Limitations and future directions

Findings from the current study should be considered in the context of several limitations. First, the sample size is notably small. While the current study utilized a sample almost double that of prior imaging research in this area, efforts to examine potential differences between subgroups of youth with CP resulted in relatively small group sizes and could undermine the ability to detect effects. Moreover, analyses examining associations between reward/punishment processing and treatment responsiveness were notably exploratory in nature given the small number of CP youth who completed both an fMRI scan and treatment. Thus, null findings may be attributable to a lack of power. Second, the current study utilized a task designed to examine the BOLD response to *receipt* of reward and punishment in attempt to clarify discrepancies in the extant literature and isolate potential functional abnormalities during this specific phase of learning. It is possible that individual differences in reward and/or punishment processing may be evident or more pronounced during different phases of learning (i.e., anticipation, acquisition, extinction) and future research is needed to address these questions. Third, in line with the new DSM-5 specifier, CU traits were measured using 4-items from the APSD, a measure that has been noted for its lack of internal consistency (Munoz and Frick, 2007). While emerging research suggests that utilizing extreme responses on 4 similar items provided the best discrimination in IRT analyses and delineated community youth who were highly antisocial (Kimonis et al., 2015), future studies may seek a more comprehensive assessment (e.g., different measures and informants), consistent with DSM-5 recommendations. Finally, the current study utilized in a clinical sample of primarily African-American boys with severe CP in late-childhood, limiting the degree to which these results can be generalized to community samples, girls and/or adolescents.

In sum, the current study offers new insight into the characterization of abnormalities in reward/punishment processing in CP youth, as it is the first known investigation to examine these mechanisms among subgroups of CP youth with high versus low CU traits. Findings highlight reduced amygdala activation to punishment among youth with early-onset CP and suggest these deficits are *not* specific to CP youth with high CU traits. Importantly, these abnormalities were not associated with treatment response and instead may represent potential avenues for more individualized approaches to intervention.

## Disclosure

All authors report no biomedical financial interests or potential conflicts of interest.

## Footnotes

1 While there is no standard baseline in the field (see Galvan, 2010 for a discussion of this), the use of an implicit baseline has been validated in previous neuroimaging studies focused on reward/punishment processing in youth (e.g., Galván and McGlennen, 2013; Goff et al., 2013; Bradley et al., 2017).

2 A single mask was created using a 20 mm^3^ sphere centered on MNI coordinates *x* = 0*, y* = 14*, z* = −15, encompassing the ventral striatum and caudate head, and this was combined with the caudate body/tail and putamen specified using AAL masks in the WFU Pic-Atlas Tool (v3.0.3)

3 We used AFNI’s recently revised methodology for 3dClustsim, which is intended to provide improved estimation of FWHM smoothness values via implementation of an AutoCorrelation Function (ACF). See https://afni.nimh.nih.gov/pub/dist/doc/program_help/3dClustSim.html

4 Supplemental Table 3 depicts a full correlation matrix of all study variables and significant clusters identified in primary group analyses:1) left mPFC; 2) left amygdala; 3) left caudate. These are the same clusters utilized in repeated measures ANOVAs investigating treatment effects.

5 Results were identical when post-treatment levels of CP were examined categorically (responders versus non-responders).
