# Supplementary material for: Boys with conduct problems and callous-unemotional traits: Neural response to reward and punishment and associations with treatment response
Source: Dev Cogn Neurosci. 2017 Dec 15;30:51–9. doi: 10.1016/j.dcn.2017.12.004 (PMC5993203; doi:10.1016/j.dcn.2017.12.004)
Supplement: Supplementary file 1 [file mmc1.docx]

**Supplemental Table 1. Suprathreshold clusters associated with combined effects of large and small reward relative to baseline, collapsed across group.**

|  |  |  | Peak Coordinates (MNI) | | |  |
| --- | --- | --- | --- | --- | --- | --- |
| Region | R/L | Voxels | X | Y | Z | t |
| Posterior Cingulate Gyrus, Insula, Thalamus, Striatum | R/L | 1129 | 3 | -28 | 28 | 10.86 |
| Middle Frontal Gyrus | R | 829 | 50 | 9 | 34 | 9.99 |
| Inferior Parietal Lobule | R | 781 | 47 | -47 | 56 | 14.73 |
| Middle Occipital Gyrus, Fusiform Gyrus | R | 606 | 34 | -90 | -6 | 16.34 |
| Middle Occipital Gyrus | L | 501 | -28 | -94 | -3 | 12.62 |
| Superior Parietal Lobule, Precuneus, Inferior Parietal Lobule | L | 483 | -25 | -66 | 43 | 9.26 |
| Inferior Frontal Gyrus | L | 384 | -44 | 6 | 25 | 10.25 |
| Anterior Cingulate Gyrus (BA32) | R/L | 338 | 3 | 22 | 43 | 10.32 |
| Middle Frontal Gyrus | L | 54 | -28 | 6 | 59 | 6.39 |
| Parahippocampal Gyrus | L | 47 | -22 | -31 | -3 | 6.82 |
| Cerebellum, Occipital Lobe | L | 43 | -16 | -72 | 9 | 6.72 |

*Note*. R=right; L=left; BA=Brodmann’s area.

# Supplemental Table 2. Suprathreshold clusters associated with combined effects of large and small punishment relative to baseline, collapsed across group.

|  |  |  | Peak Coordinates (MNI) | | |  |
| --- | --- | --- | --- | --- | --- | --- |
| Region | R/L | Voxels | X | Y | Z | t |
| Postcentral Gyrus, Inferior Parietal Lobule | R | 405 | 53 | -37 | 53 | 9.54 |
| Middle Occipital Gyrus | R | 331 | 34 | -90 | -6 | 13.85 |
| Inferior Parietal Lobule | L | 246 | -47 | -53 | 53 | 7.91 |
| Middle & Inferior Occipital Gyrus | L | 214 | -28 | -94 | -3 | 10.76 |
| Superior Frontal Gyrus, Anterior Cingulate Gyrus | R | 202 | 3 | 16 | 53 | 8.68 |
| Thalamus | R | 106 | 3 | -9 | 9 | 8.58 |
| Inferior Frontal Gyrus | L | 104 | -41 | 3 | 31 | 7.52 |
| Middle Frontal Gyrus, Inferior Frontal Gyrus | R | 84 | 47 | 41 | 22 | 6.84 |
| Middle Frontal Gyrus | R | 77 | 50 | 12 | 31 | 7.62 |
| Middle Frontal Gyrus | R | 71 | 37 | 3 | 56 | 7.39 |
| Precuneus | R | 53 | 3 | -69 | 40 | 6.93 |
| Insula | R | 49 | 44 | 16 | -6 | 6.40 |
| Insula, Inferior Frontal Gyrus (BA47) | L | 25 | -37 | 16 | -3 | 6.52 |
| Cingulate Gyrus | R | 23 | 3 | -28 | 25 | 7.70 |
| Thalamus | R | 21 | 22 | -34 | 0 | 6.46 |

*Note*. R=right; L=left; BA=Brodmann’s area.

**Supplemental Table 3. Correlations for all study variables and primary outcomes.**

|  | 1 | 2 | 3 | 4 | 5 | 6 | 7 | 8 | 9 | 10 | 11 |
| --- | --- | --- | --- | --- | --- | --- | --- | --- | --- | --- | --- |
| 1. Age |  |  |  |  |  |  |  |  |  |  |  |
| 2. Race (African-American) | 0.04 |  |  |  |  |  |  |  |  |  |  |
| 3. Public Assistance (yes/no) | 0.04 | 0.07 |  |  |  |  |  |  |  |  |  |
| 4. IQ | -0.10 | **-0.28*** | -0.20 |  |  |  |  |  |  |  |  |
| 5. ADHD symptoms | 0.16 | 0.13 | 0.06 | **-0.33^**^** |  |  |  |  |  |  |  |
| 6. Internalizing symptoms | 0.16 | 0.04 | -0.02 | **-0.34^**^** | **0.64^**^** |  |  |  |  |  |  |
| 7. Conduct Problems | 0.09 | 0.23 | 0.05 | **-0.40^**^** | **0.78^**^** | **0.68^**^** |  |  |  |  |  |
| 8. APSD CU Traits | 0.08 | 0.02 | 0.06 | -0.06 | **0.58^**^** | **0.41^**^** | **0.58^**^** |  |  |  |  |
| 9. APSD Total Score | 0.12 | 0.01 | 0.07 | -0.23 | **0.69^**^** | **0.55^**^** | **0.73^**^** | **0.83^**^** |  |  |  |
| 10. Left mPFC (8 voxels) | -0.14 | 0.10 | -0.06 | 0.15 | **-0.33^**^** | **-0.33^**^** | **-0.34^**^** | -0.12 | -0.22 |  |  |
| 11. Left Amygdala (22 voxels) | -0.18 | -0.04 | 0.00 | 0.12 | **-0.43^**^** | **-0.39^**^** | **-0.45^**^** | **-0.39^**^** | **-0.43^**^** | **0.64^**^** |  |
| 12. Left Caudate (14 voxels) | -0.02 | 0.06 | 0.20 | 0.00 | -0.06 | -0.06 | -0.05 | 0.13 | -0.05 | **0.51^**^** | **0.55^**^** |

Table depicts a full correlation matrix of all study variables and significant clusters identified in primary group analyses.

*Note.* ADHD=attention deficit hyperactivity disorder; APSD=Antisocial Process Screening Device; IQ=intelligent quotient; mPFC= medial prefrontal cortex.

* = *p <* 0.05; ** = *p <* 0.01, significant effects are bolded.

**Supplemental Table 4. Results from three separate repeated measures ANOVAs investigating the effect treatment group, BOLD response and their interaction on within individual changes in conduct problem severity from baseline to post-treatment.**

|  | Time X TX Group | Time X  BOLD response | Time X  TX Group X BOLD response |
| --- | --- | --- | --- |
| 1. Left mPFC (8 voxels) | 8.16** | 0.20 | 1.18 |
| 2. Left Amygdala (22 voxels) | 5.44* | 0.70 | 0.07 |
| 3. Left Caudate (14 voxels) | 8.55** | 0.96 | 2.26 |

A separate repeated-measures ANOVA was conducted for each of the significant clusters identified in the primary group (pre-treatment) analyses. Each enumerated row represents a separate repeated-measures ANOVA with one within-person factor (time) to reflect within-individual differences in CP from baseline to the 3-month post-treatment assessment, and two between-person factors (treatment condition, BOLD response). Of interest for the current analyses were the following cross-level interactions: 1) time by treatment group; 2) time by BOLD response; and 3) time by treatment group by BOLD response.

*Note.* BOLD=blood oxygen level dependent; mPFC=medial prefrontal cortex; TX=treatment; X=interaction.

* = *p <* 0.05; ** = *p <* 0.01

*a.*

**
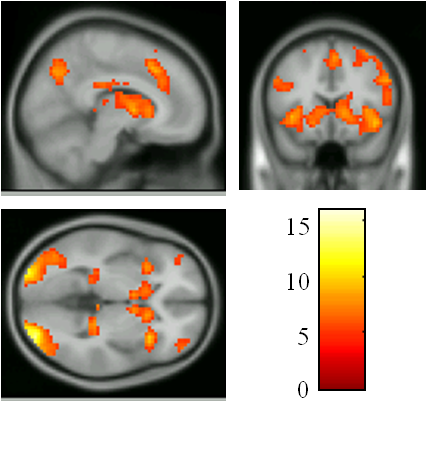
**

*b.*

**
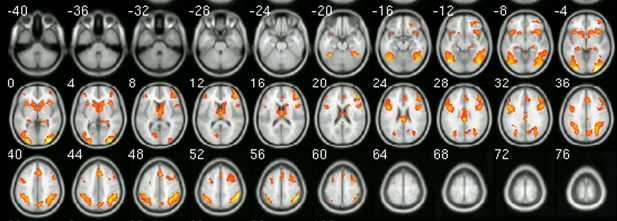
**

# Supplemental Figure 1. Combined effects of large and small reward reveal robust task-related activation throughout reward-related circuitry.

Overall task-related activation associated with large and small reward compared with implicit baseline, collapsed across group, with whole-brain family-wise error (FWE) correction of *p <* 0.05, 20 voxel extent threshold. a) Slices shown at x = 8, y = 16, z = -1. Color bar reflects t-values; b) Transverse slices shown every 4 units on the y-axis.

*a.*

*b.***
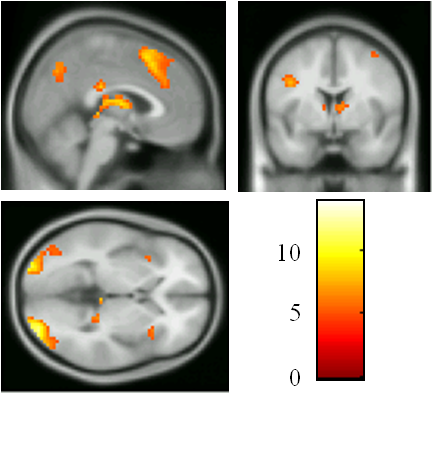
**

**
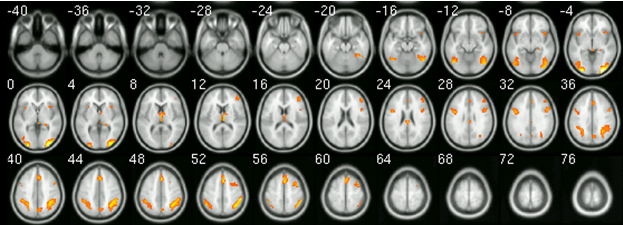
**

# Supplemental Figure 2. Combined effects of large and small punishment reveal task-related activation throughout punishment-related circuitry.

Overall task-related activation associated with large and small punishment compared with implicit baseline, collapsed across groups, with whole-brain family-wise error (FWE) correction of *p <* 0.05, 20 voxel extent threshold. a) Slices shown at x = 3 y =1, z = -1. Color bar reflects t-values; b) Transverse slices shown every 4 units on the y-axis.

#

**
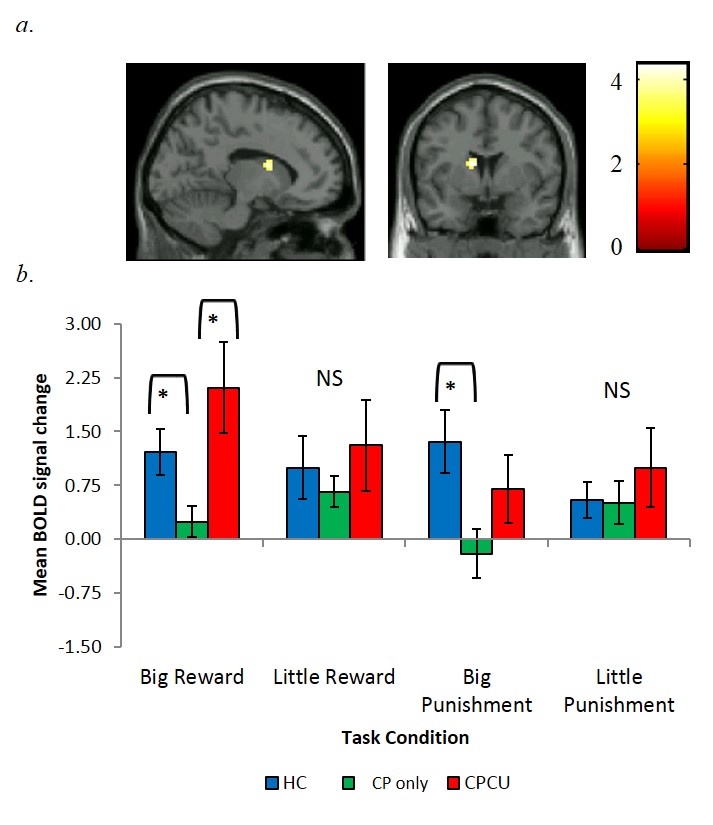
**

# Supplemental Figure 3. Interaction between group and task condition in the left caudate.

*a*. Region in the caudate significant at *p <* 0.005, corrected using 3DclusterSim threshold for contiguous voxels (*F*(6,244) = 4.37, 14 voxels). Slices shown at x = -12, y =3, z =15 (MNI peak voxel). Color bar reflects t-values; *b*. Bar graphs depict extracted mean BOLD signal change (%) across all voxels within the cluster along with standard errors.

* = *p <* 0.05. The *p*-values are based on Games-Howell pairwise comparisons for extracted mean BOLD response.

*Note*. BOLD=blood oxygen level dependent; CP=conduct problems; CU=callous-unemotional traits; HC=healthy control; NS=non-significant.
